# Glypican-3 induces oncogenicity by preventing IGF-1R degradation, a process that can be blocked by Grb10

**DOI:** 10.18632/oncotarget.19035

**Published:** 2017-07-06

**Authors:** Wei Cheng, Po-Chun Huang, Hsiao-Mei Chao, Yung-Ming Jeng, Hey-Chi Hsu, Hung-Wei Pan, Wuh-Liang Hwu, Yu-May Lee

**Affiliations:** ^1^ Department of Pathology, Kee-Lung Hospital, Ministry of Health and Welfare, Kee-Lung, Taiwan; ^2^ Ching Kuo Institute of Management and Health, Kee-Lung, Taiwan; ^3^ National Taipei University of Nursing and Health Sciences, Taipei, Taiwan; ^4^ Institute of Biochemical Sciences, National Taiwan University, Taipei, Taiwan; ^5^ Institute of Biological Chemistry, Academia Sinica, Taipei, Taiwan; ^6^ Department of Pathology, Wang Fang Hospital, Taipei Medical University, Taipei, Taiwan; ^7^ Graduate Institute of Pathology, College of Medicine, National Taiwan University, Taipei, Taiwan; ^8^ Department of Medical Education and Research, Kaohsiung Veterans General Hospital, Kaohsiung, Taiwan; ^9^ Department of Medical Genetics, National Taiwan University Hospital, Taipei, Taiwan

**Keywords:** glypican-3, hepatocellular carcinoma, insulin-like growth factor 1 receptor, ubiquitination, growth factor receptor-bound protein 10

## Abstract

Hepatocellular carcinoma (HCC) is the most common primary liver malignancy and is a major cause of cancer-related death worldwide. Previously, we demonstrated that glypican-3 (GPC3) is highly expressed in HCC, and that GPC3 induces oncogenicity and promotes the growth of cancer cells through IGF-1 receptor (IGF-1R). In the present study, we investigated the mechanisms of GPC3-mediated enhancement of IGF-1R signaling. We demonstrated that GPC3 decreased IGF-1-induced IGF-1R ubiquitination and degradation and increased c-Myc protein levels. GPC3 bound to Grb10, a mediator of ligand-induced receptor ubiquitination, and the overexpression of Grb10 blocked GPC3-enhanced IGF-1-induced ERK phosphorylation. GPC3 promoted the growth of NIH3T3 and PLC-PRF-5 cells in serum-free medium but did not promote the growth of IGF-1R negative R- cells. Grb10 overexpression decreased GPC3-promoted cell growth. Therefore, the present study elucidates the mechanisms of GPC3-induced oncogenicity, which may highlight new strategies for the treatment of HCC.

## INTRODUCTION

Hepatocellular carcinoma (HCC) is the leading cause of cancer-related death in Asia, and the incidence of HCC is increasing in many other countries [1, 2]. The molecular mechanisms for hepatocarcinogenesis are complex [3], and despite early detection and aggressive therapies, the outcome of HCC remains grave. We previously discovered that glypican-3 (GPC3, also known as MXR7) is overexpressed in HCC and is correlated with high alpha-fetoprotein, high tumor grade, and high tumor aggressiveness [4]. The overexpression of GPC3 was later observed in a variety of cancers, including metastatic colorectal carcinomas [5], alpha-fetoprotein-producing gastric carcinoma [6], hepatoblastoma [7], Wilms’ tumor [7], malignant melanoma [8], yolk sac tumor, choriocarcinoma [9], ovarian carcinoma [10], and pancreatic ductal adenocarcinoma [11]. Currently, GPC3 expression has been proved to be a poor prognostic factor for HCC [12–14].

Glypicans are a family of heparan sulfate proteoglycans (HSPGs) linked to the exocytoplasmic surface of the plasma membrane through a glycosylphos phatidylinositol (GPI) anchor [15]. Glypicans act as co-receptors by facilitating the formation of ligand-receptor complexes and effectively lowering the required concentration of ligands [16]. Loss-of-function mutations in GPC3 cause Simpson-Golabi-Behmel syndrome, a disease that is characterized by prenatal and postnatal overgrowth and an increased risk of embryonal tumor development [17]. In transgenic mice, GPC3 suppresses hepatocyte proliferation [18]. However, studies have also demonstrated that GPC3 plays a role in the development of cancer [15, 19, 20]. GPC3 regulates the signaling activity of various morphogens, including Wnts, Hedgehogs (Hhs), bone morphogenic proteins (BMPs), and fibroblast growth factors (FGFs) [21–23]. GPC3 promotes the *in vitro* and *in vivo* growth of HCC cells by interacting with the Wnt ligand to facilitate Wnt/Frizzled [19], and an antibody (HS20) against the heparan sulfate of GPC3 blocks Wnt signaling and HCC growth [24]. GPC3 may be both a serum marker [25] and a therapeutic target of HCC [26–28].

GPC3 also binds to Insulin-like growth factor-II (IGF-2) [17, 29]. IGF-1 binds to IGF-1R, while IGF-2 binds to both IGF-1R and IGF-2R, and the IGF-signaling pathway plays a pivotal role in cell proliferation [30], G1 cell cycle progression [31], prevention of apoptosis [32], and the initiation and maintenance of oncogenesis [33]. We have previously demonstrated that GPC3 binds to IGF-2 and IGF-1R through its N-terminal proline-rich domain, induces the phosphorylation of IGF-1R and extracellular signal-regulated kinase (ERK), and induces oncogenicity [20]. Increased IGF-2 expression has been observed in HCC [34], and IGF-1R is frequently overexpressed in breast cancer, thyroid cancer, melanoma, and HCC [35–38].

In the present study, we investigated the mechanisms of GPC3-mediated enhancement of IGF-1R signaling. We demonstrated that GPC3 decreased IGF-1-induced IGF-1R ubiquitination and degradation, possibly through the interaction between GPC3 and Grb10.

## RESULTS

### Expression of GPC3 and IGF-1R in HCC

**Figure 1:**
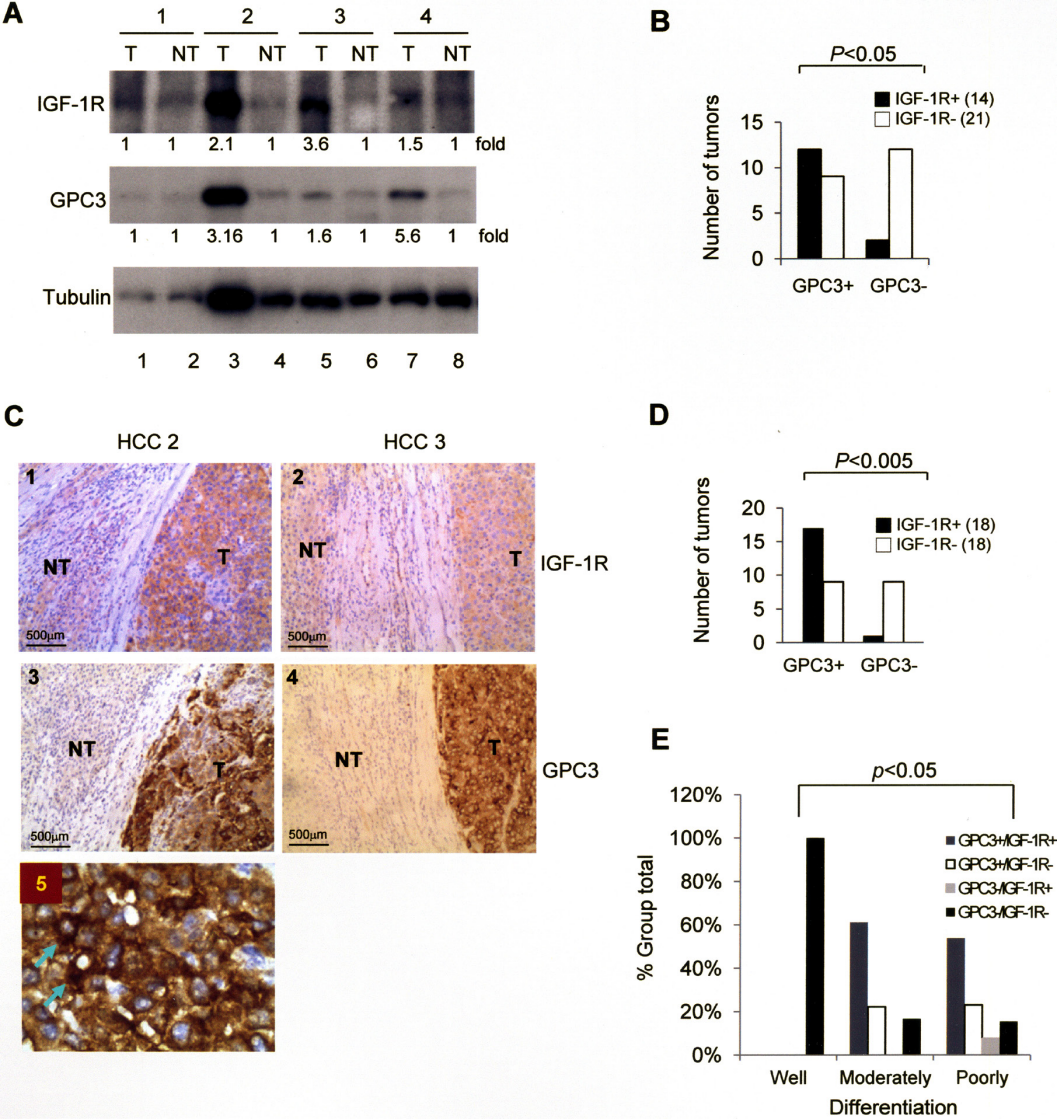
The expression of GPC3 and IGF-1R is correlated in HCC (**A**) Western blot analysis of IGF-1R and GPC3 in tumor (T) and non-tumor (NT) regions of HCC. In four representative cases, three cases (numbers 2, 3, and 4) exhibited elevated expression of both IGF-1R and GPC3. (**B**) Western blot analysis showing the correlation between GPC3 and IGF-1R expression in 35 HCC cases. (**C**) Immunohistochemistry (IHC) of GPC3 and IGF-1R in two representative HCC specimens exhibited positive GPC3 and IGF-1R staining in the tumor regions (100×). (**D**) IHC showing the correlation between GPC3 and IGF-1R expression in 36 HCC cases. (**E**) IHC showing the correlation between GPC3 and IGF-1R expression and grade of tumor differentiation in 36 HCC cases. The *p* value was determined using Fisher's exact test. The quantitation after normalization based on the quantity of tubulin was also expressed as a fold-change relative to the control experiment. All experiments were duplicated.

We examined the expression of GPC3 and IGF-1R in HCC specimens. Western blot analysis of 35 HCCs revealed a positive GPC3 signal in 21 and a positive IGF-1R signal in 14 (Figure 1A), and the presence of GPC3 and IGF-1R was correlated (Figure 1B; *p <* 0.05 by Fisher's exact test). Immunohistochemistry studies of these 36 HCCs (tumor regions) exhibited strong positive GPC3 staining in 25 and positive IGF-1R staining in 18 (Figure 1C), and the expression of GPC3 and IGF-1R was correlated (Figure 1D; *p <* 0.005 by Fisher's exact test). The non-tumor regions of all HCCs stained negative for GPC3 and IGF-1R. When we classified these 36 HCCs into well, moderately, and poorly differentiated tumors, positive staining for either GPC3 or IGF-1R was only observed in moderately and poorly differentiated HCC (Figure 1E), and therefore the expression of GPC3 and IGF-1R was correlated with tumor grade (*p <* 0.05 by Pearson's Chi-Squared test). In an amplified view of immunohistochemistry for GPC3, cytoplasmic staining of GPC3 was observed (Arrows, Figure 1C).

### GPC3 decreases IGF-1-induced IGF-1R degradation

**Figure 2:**
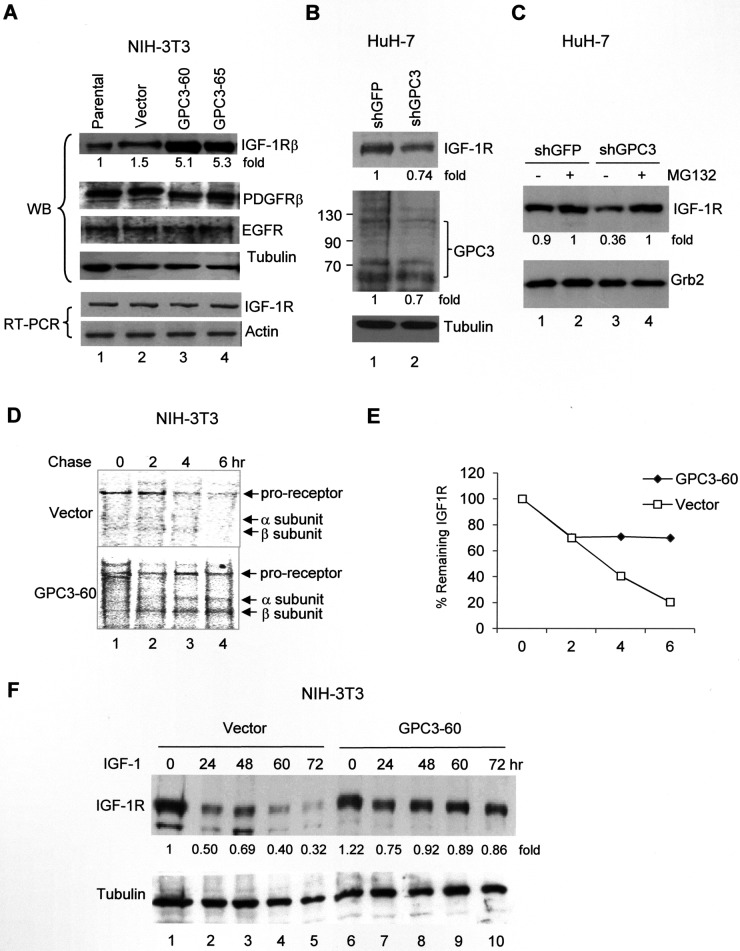
GPC3 decreases IGF-1-induced IGF-1R ubiquitination and degradation (**A**) Western blot analysis of IGF-1R in either parental NIH3T3 cells or stable clones overexpressing the vector alone or GPC3 (GPC3-60 and GPC3-65). Protein extracts (30 μg) were subjected to analysis with anti-IGF-1Rβ, PDGFRβ, EGFR, or tubulin antibodies. IGF-1R RNA levels were measured using RT-PCR, and actin was used as an internal control. (**B**) GPC3 knockdown decreases IGF-1R levels. HuH-7 cells were transfected with either shGFP or shGPC3. Western blot analysis revealed a decrease in both GPC3 and IGF-1R by shGPC3. The amount of tubulin was used as a control. (**C**) The proteasome inhibitor MG132 prevents IGF-1R degradation. HuH-7 cells were transfected with either shGFP or shGPC3 and treated with MG132. In the presence of MG132, shGPC3 could not decrease IGF-1R levels. The amount of Grb2 was used as a control. (**D**) Pulse-chase study. GPC3-60 cells were serum starved, pulsed with [^35^S]Cys-Met, stimulated with IGF-1, and chased for up to 6 hours. Immunoprecipitation was performed with an anti-IGF-1Rβ antibody. (**E**) Quantitation of the IGF-1R levels shown in panel (D) using densitometric analysis. (**F**) A time-course study for IGF-1-induced IGF-1R degradation. NIH3T3 stable lines overexpressing the vector alone or GPC3 (GPC3-60) were harvested for western blot analyses 0, 24, 48, 60, and 72 hrs after IGF-1 stimulation. The degradation of IGF-1R was prevented through the overexpression of GPC3 (*p <* 0.05, Fisher's exact test). Tubulin was used as a control. Quantitation after normalization by the quantity of tubulin or Grb2 was also expressed as a fold-change relative to the control experiment. All experiments were duplicated.

We next asked whether GPC3 was the etiology of IGF-1R overexpression in HCC. In the GPC3-overexpressing NIH3T3 clones GPC3-60 and 65, IGF-1R expression was elevated (Figure 2A; *p <* 0.005, *T* test), but not the levels of other growth factor receptors, including platelet-derived growth factor receptor (PDGFR) and epidermal growth factor receptor (EGFR). The RNA levels of IGF-1R were not changed, indicating that the elevation of the quantity of IGF-1R protein was not due to gene transcription activation (Figure 2A). By contrast, we knocked down GPC3 expression in HuH-7 cells using shGPC3. Although GPC3 levels were only slightly decreased because many cells died after treatment [20], the levels of IGF-1R were also decreased (Figure 2B, lane 2; *p <* 0.005, *T* test). When the cells were pretreated with the proteasome inhibitor MG132 and then subjected to shRNA, the IGF-1R levels were elevated by MG132, but the effects of shGPC3 were lost (Figure 2C, lane 4). We subsequently performed a pulse-chase study to measure the rate of IGF-1R degradation after IGF-1 stimulation. The results revealed that the labeled IGF-1R proreceptor, which is processed into α and β subunits, exhibited a half-life of 3 hrs in control cells but a half-life longer than 6 hrs in GPC3-60 cells (Figure 2D and 2E; *p <* 0.05 at 4 hr and 6 hr, *T* test). In a time-course study in control NIH3T3 cells, the levels of IGF-1R decreased dramatically after IGF-1 stimulation (Vector, Figure 2F). However, in GPC3 over-expressing GPC3-60 cells, IGF-1R levels only slightly decreased at 24 hrs after IGF-1 stimulation and remained stable thereafter (GPC3, Figure 2F; *p <* 0.05, Fisher's exact test). In the control study, a small rebound in IGF-1R levels was observed at 48 hrs (Lane 3, Figure 2F). This phenomenon has previously been reported [39] and likely occurred in the present study because we did not add cycloheximide (CHX) to block translation elongation.

### GPC3 prevents IGF-1-induced IGF-1R ubiquitination

**Figure 3:**
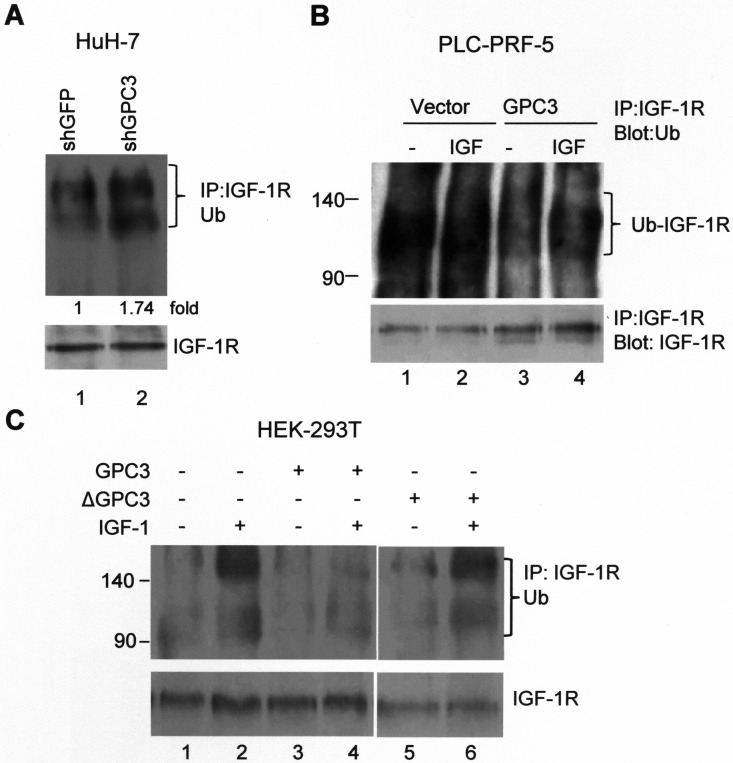
GPC3 decreases IGF-1R ubiquitination (**A**) IGF-1R ubiquitination increased after GPC3 knockdown in HuH-7 cells. The cells were transfected with shGFP or shGPC3 and subsequently cultured in serum-free medium for 1 day. Immunoprecipitation was performed with anti-IGF-1Rβ, and western blot analysis was performed with anti-ubiquitin. Increased IGF-1R ubiquitination was observed in GPC3 knockdown cells (lane 2; *p <* 0.001, *T* test). Quantitation was also expressed as a fold-change relative to the control experiment. (**B**) IGF-1R ubiquitination decreased after GPC3 overexpression in PLC-PRF-5 cells. PLC-PRF-5 stable clones expressing either an empty vector or GPC3 were stimulated with IGF-1, immunoprecipitation was performed using anti-IGF-1Rβ, and western blot analysis was performed using anti-ubiquitin. IGF-1R ubiquitination levels were high in PLC-PRF-5 cells, but this increase was prevented through the overexpression of GPC3 (lane 3 and 4) with or without IGF-1 stimulation. (**C**) HEK293T cells were transfected with GPC3 or deleted GPC3 (ΔGPC3), together with ubiquitin, Grb10, and Nedd4. The cells were stimulated with IGF-1, and subsequently immunoprecipitation was performed using anti-IGF-1Rβ, and western blot analysis was performed using anti-ubiquitin. IGF-1 stimulation induced the ubiquitination of IGF-1R (lane 2), which was prevented by the overexpression of GPC3 (lane 4), but not ΔGPC3 (lane 6). The amount of IGF-1R was slightly decreased by ΔGPC3. All experiments were duplicated.

Ligand-induced receptor degradation is mediated through the ubiquitination of the receptor, followed by lysosome or proteasome degradation. We explored whether GPC3 affected the dynamics of IGF-1R ubiquitination. In HuH-7 cells, treatment with shGPC3 increased the levels of ubiquitinated IGF-1R (Figure 3A; *p <* 0.001, *T* test). In PLC-PRF-5 cells, the overexpression of GPC3 decreased the levels of ubiquitinated IGF-1R compared with the control cells both before and after IGF-1 stimulation (Figure 3B). We next induced IGF-1R ubiquitination in HEK293T cells through the transfection of Grb10, Nedd4, and ubiquitin into these cells. The Grb10 (growth factor receptor-bound protein 10) and Nedd4 (neural precursor cell-expressed developmentally downregulated protein 4) complex is known to mediate ligand-induced ubiquitination of IGF-1R [40]. After IGF-1 stimulation, immunoprecipitation was performed using anti-IGF-1Rβ, and western blot analysis was performed using anti-ubiquitin. The results showed that IGF-1 stimulation triggered the ubiquitination of IGF-1R (Figure 3C, lane 2), which was prevented by the overexpression of GPC3 (Figure 3C, lane 4) but not ΔGPC3 (GPC3 with deleted 70 a.a. to 453 a.a.; Figure 3C, lane 6).

### GPC3 enhances of ERK phosphorylation and c-Myc expression

**Figure 4:**
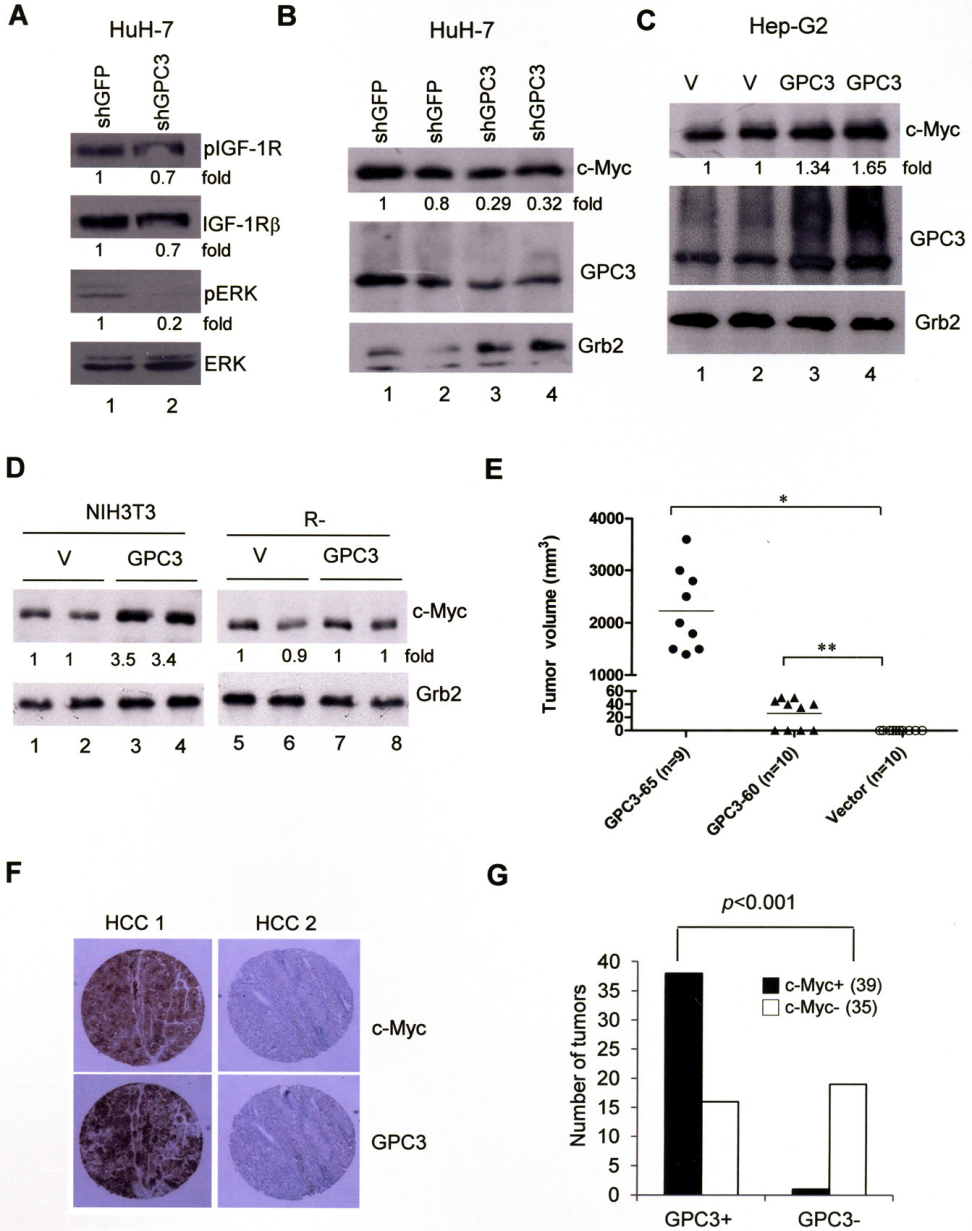
ERK phosphorylation and c-Myc expression are both dependent on GPC3 (**A**) HuH-7 cells were transiently transfected with shGFP or shGPC3. The cells were subjected to western blot analysis for phospho-IGF-1R, IGF-1R, phospho-ERK, and ERK. ERK phosphorylation was decreased by GPC3 knockdown (*p <* 0.005, *T* test). (**B**) HuH-7 cells were transiently transfected with shGFP or shGPC3. The cells were subjected to western blot analysis for c-Myc. The levels of c-Myc were reduced by GPC3 knockdown (*p <* 0.02, *T* test). (**C**) HepG2 cells were transfected with either GPC3 or an empty vector (V). Western blot analysis was performed for GPC3 and c-Myc. The levels of c-Myc were increased by the overexpression of GPC3 (*p <* 0.05, *T* test). The quantity of Grb2 was used as a control. (**D**) c-Myc expression was increased by GPC3 expression in NIH3T3 cells (*p <* 0.001, *T* test), but not in R- cells. Grb2 was used as a loading control. Quantitation after normalization was also expressed as a fold-change relative to the control experiment. (**E**) Dissected tumor volume isolated from GPC3-65 (*n* = 9) and GPC3-60 injected mice (*n* = 10). No tumor growth was observed in mice injected with vector control cells (*n* = 10) (*p < 0.001; **p < 0.005, *T* test). (**F**) Immunohistochemical staining of GPC3 and c-Myc in HCCs. HCC 1 showed the positive staining of GPC3 and c-Myc in tumor regions. HCC 2 showed the negative staining of either GPC3 or c-Myc. (**G**) Results of immunohistochemistry analysis of 74 HCC specimens for GPC3 and c-Myc. The expression of GPC3 and c-Myc was highly correlated (*p* < 0.001, Fisher's exact test). All experiments were duplicated.

The ligation of IGF-1R is known to activate the ERK and c-Myc signal transduction pathway. In HuH-7 cells, GPC3 knock down by shGPC3 decreased ERK phosphorylation (Figure 4A, lane 2; *p <* 0.005, *T* test) and the levels of c-Myc (Figure 4B; *p <* 0.02, *T* test). The overexpression of GPC3 increased the levels of c-Myc in HepG2 cells (Figure 4C; *p <* 0.05, *T* test) and NIH3T3 cells (Figure 4D; *p <* 0.001, *T* test) but not in IGF-1R-negative R- cells (Figure 4D). These data suggest that GPC3 enhances ERK phosphorylation and c-Myc expression through IGF-1R. When we injected NIH3T3 cells into nude mice, no tumor growth was observed. However, the injection of GPC3-60 and GPC3-65 cells generated tumors in the mice (Figure 4E), while the injection of GPC3-expressing R- cells did not (data not shown). We therefore examined the expression of c-Myc in HCCs using immunohistochemistry. Among 74 HCCs, 39 were positive for c-Myc, among which 38 of the 39 c-Myc-positive HCCs were also positive for GPC3 (Figure 4F). Therefore, the expression of GPC3 is correlated with that of c-Myc (*p <* 0.001, Fisher's exact test; Figure 4G).

### Grb10 binds to GPC3

**Figure 5:**
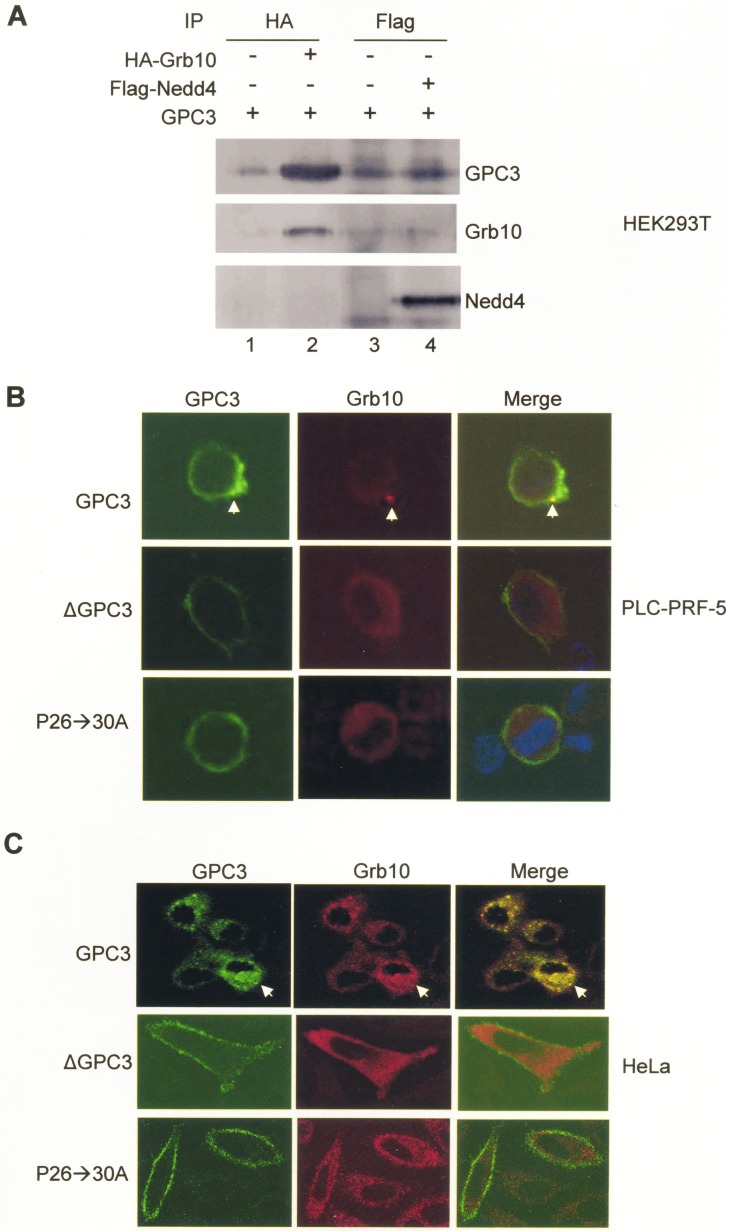
Grb10 binds to GPC3 (**A**) HEK293T cells were transfected with GPC3 together with HA-Grb10 or Flag-Nedd4. Immunoprecipitation performed with anti-HA or anti-Flag demonstrated that GPC3 was pulled down by Grb10 (lane 2), but not Nedd4 (lane 4). (**B** and **C**) Colocalization of GPC3 and Grb10. PLC-PRF-5 (B) and HeLa (C) cells were transfected with IGF-1R and Grb10, together with wild-type GPC3, ΔGPC3, or P26->30A and subsequently stimulated with IGF-1. Immunofluorescence staining was performed for GPC3 (green) and Grb10 (red). Wild type GPC3, but not ΔGPC3 or P26->30A was internalized and colocalized with Grb10 (arrow). All experiments were duplicated.

We further explored the mechanisms underlying the modulation of IGF-1-induced IGF-1R ubiquitination by GPC3. Grb10 and Nedd4 are mediators of ligand-induced receptor ubiquitination; thus, we explored interactions between GPC3 and Grb10 or Nedd4. In HEK293T cells co-transfected with GPC3 and Grb10 or Nedd4, immunoprecipitation studies showed that GPC3 was pulled down by Grb10 (Figure 5A, lane 2) but not by Nedd4 (Figure 5A, lane 4). In PLC-PRF-5 and HeLa cells transfected with IGF-1R, Grb10, and GPC3, immunofluorescence staining demonstrated that only wild-type GPC3, but not ΔGPC3 or the proline to alanine GPC3 mutant P26->30A (previously designated P25->29A) [20], was internalized and colocalized with Grb10 after IGF-1 stimulation (Arrows, Figure 5B and 5C).

### Grb10 blocks GPC3-enhanced IGF-1-induced ERK phosphorylation

**Figure 6:**
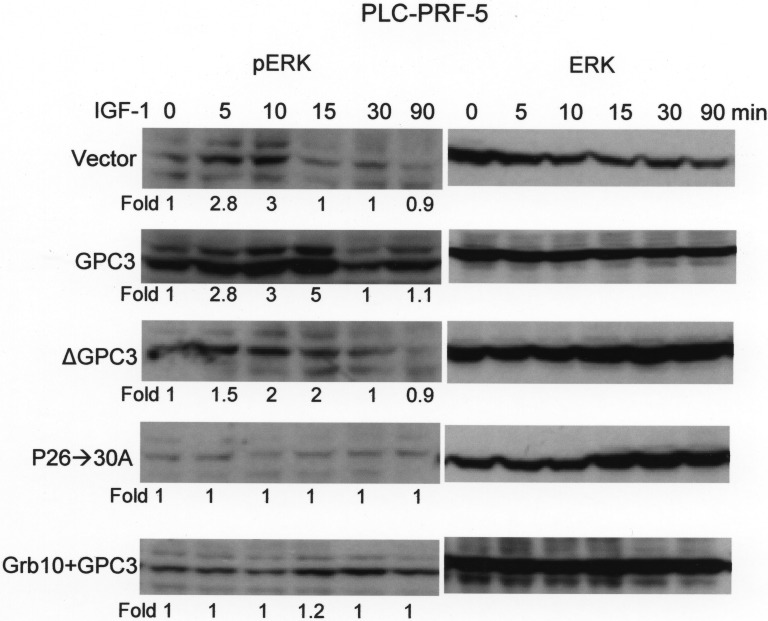
Grb10 blocks GPC3-enhanced IGF-1-induced ERK phosphorylation PLC-PRF-5 cells were transfected with GPC3, ΔGPC3, P26->30A, or Grb10 + GPC3, and stable clones were selected. Western blot analysis for ERK and phosphor ERK (pERK) was performed at 0, 5, 10, 15, 30, and 90 min after IGF-1 treatment. In cells expressing empty vector (Vector), the phosphorylation of ERK occurred shortly after IGF-1 stimulation (*p <* 0.001 at 15 min, *T* test) but decreased 15 min later. GPC3 overexpression resulted in exaggerated and prolonged ERK phosphorylation. ΔGPC3 and GPC3 mutant P26->30A did not show exaggerated ERK phosphorylation. The overexpression of Grb10 blunted IGF-1-induced ERK phosphorylation and blocked the effects of GPC3 overexpression. All experiments were duplicated.

PLC-PRF-5 cells, which expressed low levels of endogenous GPC3, were first transfected with GPC3, ΔGPC3, P26->30A, or Grb10 + GPC3 and selected for stable clones using G418. ERK phosphorylation was subsequently measured at 0, 5. 10, 15, 30, and 90 min after IGF-1 stimulation. In cells expressing the empty vector (Vector), the phosphorylation of ERK occurred shortly after stimulation but decreased 15 min later (Figure 6; *p <* 0.001 at 15 min, *T* test). The overexpression of GPC3 not only exaggerated the phosphorylation of ERK but also prolonged the duration of phosphorylation. ΔGPC3 and P26->30A did not exaggerate ERK phosphorylation. However, the overexpression of Grb10 blocked the GPC3-mediated enhancement of ERK phosphorylation.

### Grb10 decreases GPC3- and IGF-1R-dependent oncogenicity

**Figure 7:**
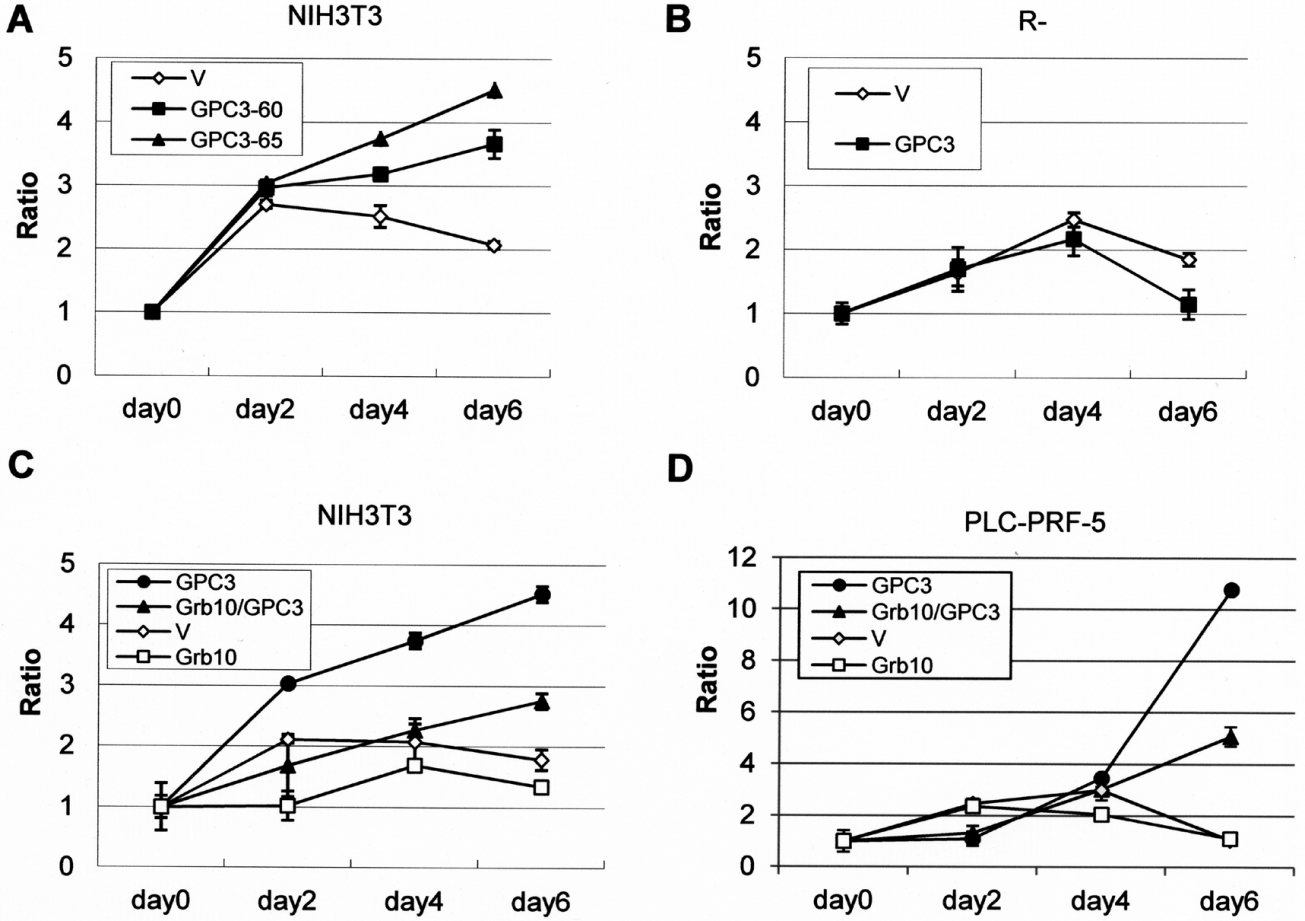
Grb10 decreases GPC3- and IGF-1R-dependent oncogenicity The cells were transfected with expression vectors, and stable clones were grown in serum-free or low-serum medium for 6 days. Fold-cell number multiplication (Ratio) is depicted. (**A**) GPC3-expressing NIH3T3 clones grew better than empty vector (V)-expressing cells in serum-free medium. (*p <* 0.005 for GPC3-60 and *p <* 0.001 for GPC-65, *T* test). (**B**) GPC3-expressing R- cells grew poorer than the empty vector-expressing R- cells in 0.5% serum (*p <* 0.005, *T* test). (**C**) The overexpression of Grb10 decreased the growth rate of GPC3-expressing NIH3T3 cells in serum-free medium (*p <* 0.001, *T* test). (**D**) The overexpression of Grb10 decreased the growth rate of GPC3-expressing PLC-PRF-5 cells in serum-free medium (*p <* 0.005, *T* test). All experiments were duplicated.

NIH3T3 cells do not exhibit growth in serum-free medium or tumorgenicity in nude mice, but GPC3-expressing NIH3T3 clones exhibited growth in serum-free medium (Figure 7A). However, GPC3-expressing IGF-1R-negative R- cells showed poorer growth in low-serum medium (Figure 7B). More interestingly, Grb10 overexpression decreased the GPC3-promoted growth of both NIH3T3 cells (Figure 7C) and PLC-PRF-5 cells (Figure 7D) in serum-free medium.

## DISCUSSION

**Figure 8:**
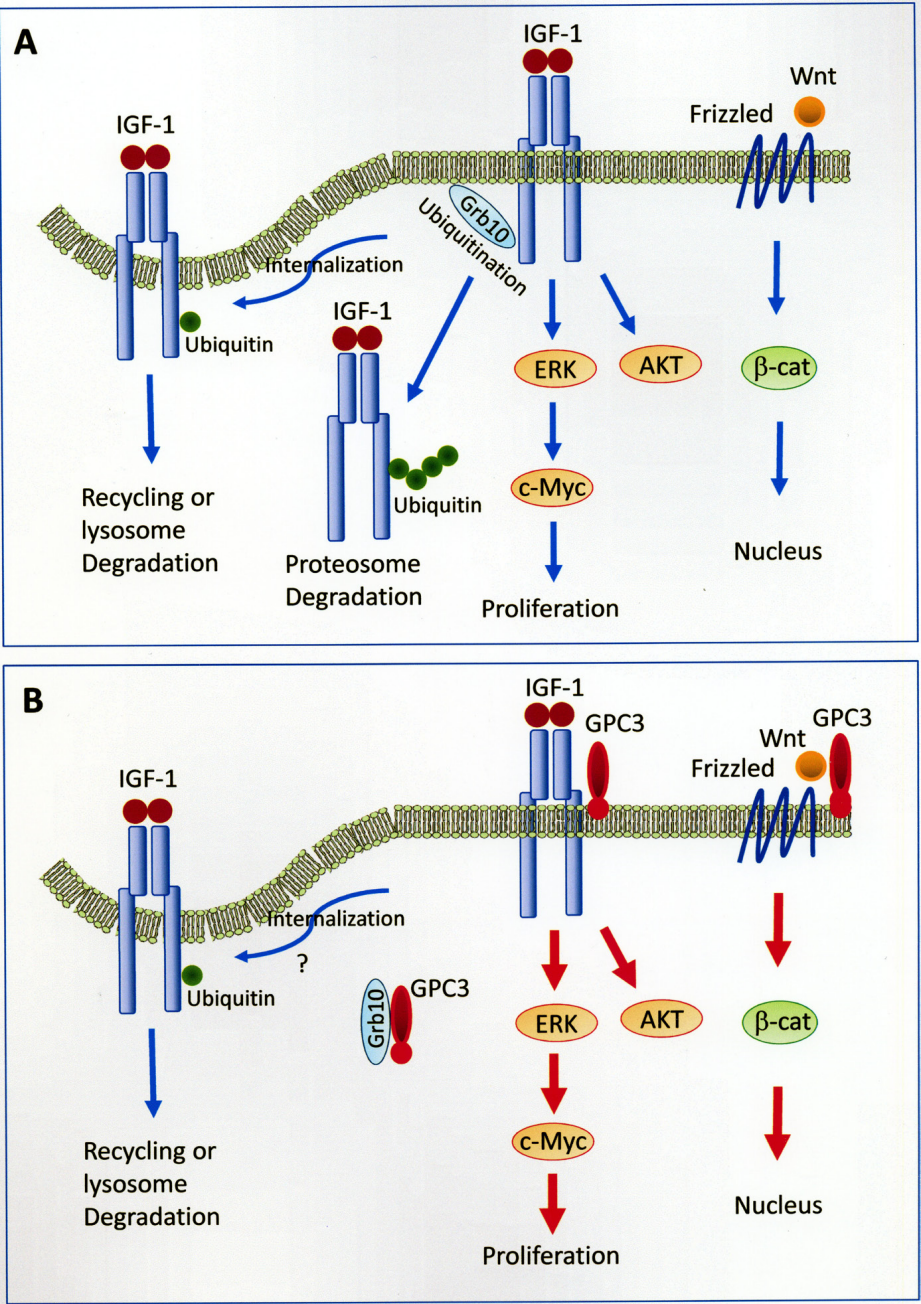
Models of GPC3-enhanced signal transduction (**A**) Activation of Wnt and IGF-1 signaling pathways without the presence of GPC3. Frizzled serves as a cell-surface receptor for Wnt, and the canonical Wnt pathway leads to the accumulation of β-catenin in the cytoplasm and its eventual translocation into the nucleus to act as a transcriptional coactivator. The binding of IGF-1 to IGF-1R induces the activation of both ERK and c-Myc, which leads to cell proliferation, and the AKT signaling pathway, which leads to protein synthesis and growth. The binding of IGF-1 to IGF-1R also leads to receptor ubiquitination, which requires the function of Grb10. Monoubiquitination serves as a regulatory signal for receptor internalization and endosomal sorting, while polyubiquitination targets proteins for degradation by the 26S proteasome [42, 43]. The mechanism for the translocation of polyubiquitinated receptor tyrosine kinase to the proteasome is currently not clear. (**B**) GPC3 activates β-catenin [19], ERK, AKT [44], and c-Myc [45] through cell-membrane receptors. GPC3 binds to and potentially sequestrates Grb10, thereby blocking IGF-1R ubiquitination and degradation in the proteasome. The status of receptor internalization in the presence of GPC3 was not examined in the present study. The mechanism for the cytoplasmic translocation of GPC3 is currently not clear.

In the present study, we demonstrated that GPC3 enhanced IGF-1R signaling, which led to ERK phosphorylation, c-Myc expression, and increased oncogenicity, and we propose a model for this mechanism (Figure 8). GPC3 has been shown to activate the canonical Wnt pathway in 18% of HCCs [41] and the IGF-1R pathway in 65.4% of GPC3-positive HCCs (present study). Frizzled serves as a cell-surface receptor for Wnt, and the canonical Wnt pathway leads to the accumulation of β-catenin in the cytoplasm and its eventual translocation into the nucleus to act as a transcriptional coactivator. The binding of IGF-1 to IGF-1R induces the activation of both ERK and c-Myc, which leads to cell proliferation, and the AKT signaling pathway, which leads to protein synthesis and growth. The binding of IGF-1 to IGF-1R also leads to receptor ubiquitination, which requires the function of Grb10. Monoubiquitination serves as a regulatory signal for receptor internalization and endosomal sorting, while polyubiquitination targets proteins for degradation by the 26S proteasome [42, 43]. GPC3 activates β-catenin [19, 24], ERK, AKT [44], and c-Myc [45] through cell-membrane receptors. GPC3 binds to and potentially sequestrates Grb10, thereby blocking IGF-1R ubiquitination and degradation in the proteasome.

Currently, the mechanism for the cytoplasmic translocation of GPC3 is not known. GPC3 is an extracellular protein that attaches to the cell surface through a glycosylphosphatidylinositol (GPI) anchor [15]. However, in the present study, we first showed the cytoplasmic staining of GPC3 in HCC specimens (Arrows, Figure 1C) and further demonstrated the cytoplasmic translocation of GPC3 in cultured cells (Figure 5B and 5C). GPC3 was also recently shown to be effectively internalized in HCC cells in another study [46]. Grb10 is located in the cytoplasm and can be relocalized to the plasma membrane after treatment with IGF-1 [47, 48]. Therefore, GPC3 may interact with Grb10 close to the cell membrane or in the cytoplasm.

Gene amplification, fusion, gain of function mutations, and the post-transcriptional regulation of growth factors and their receptors play important roles in the development of cancers. The ubiquitination-dependent regulation of signaling receptors in cancers has been demonstrated [49]. Typically, signaling receptors undergo endocytosis, which removes these proteins from the cell surface, and an internalized receptor may subsequently be recycled back to the cell surface or targeted toward late endosomes for subsequent lysosomal degradation [50]. The ubiquitination of signaling receptors or their adaptors mediates an increase in the efficacy of proteasomal or postinternalization lysosomal degradation. The rate of ubiquitin conjugation to a receptor is determined by both the intracellular domain of a receptor to recruit a specific E3 ubiquitin ligase and the overall activity of such a ligase. Accordingly, cancer cells promote their growth by changing the activities of specific E3 ubiquitin ligases or by adjusting the affinity of receptors for these ligases [50, 51]. Nedd4 is an E3 ubiquitin ligase that regulates diverse biological processes [52], and Grb10 is an adaptor protein [53]. The Grb10/Nedd4 complex regulates ligand-induced ubiquitination and IGF-1R stability [40]. Grb10 negatively regulates insulin signaling [54] and Grb10 expressed from the maternal allele restricts fetal growth [55]. We now demonstrate that GPC3 binds to Grb10 to enhance IGF-1R signaling.

Different strategies to target GPC3 for the treatment of HCC are developing. Therapeutically targeting glypican-3 via a conformation-specific single-domain antibody in HCC has been reported [56]. An anti-GPC3 antibody can be fused to a fragment of Pseudomonas exotoxin A (PE38) to create immunotoxins to induce the regression of liver tumor xenografts in mice [46]. A soluble GPC3, lacking the GPI-anchoring domain, has also been shown to inhibit HCC cell growth, probably through competition with endogenous GPC3 for protein binding [44, 57]. IGF-1R blockage has also been tested as a treatment for cancers, including HCC [58], but there were serious adverse events, including dehydration, asthenia, and hyperglycemia [59, 60]. In the present study, the mechanisms of GPC3-mediated enhancement of IGF-1R are further elucidated and may be targeted to treat HCC and to improve the outcome of patients in the future.

## MATERIALS AND METHODS

### Tissues and cells

Surgically resected liver tissue specimens were obtained from the Department of Pathology, National Taiwan University Hospital (NTUH) and Kee-Lung Hospital, Ministry of Health and Welfare (KLH). These specimens were used in accordance with the regulations and IRB approval of the Ethics Committees of the NTUH and KLH (TYGH100038). HCC cell lines HepG2 (HB-8065) and PLC-PRF-5 (CRL-8024) were purchased from ATCC (VA, USA), and HuH-7 cells [61] (JCRB-0403) were purchased from JCRB (Osaka, Japan). HEK293T cells were purchased from ATCC (CRL-3216). The reauthentication of these cells by STR DNA profiling analysis was performed prior to use (please see Supplementary Materials for STR analysis reports). NIH3T3 cells (BCRC-60008) were purchased from BCRC (Hsinchu, Taiwan). NIH3T3 is a non-human cell line, and the morphology and speed of growth of these cells have not changed since they were obtained in 2003 (data not shown). Both HEK293T and NIH3T3 cells were used for transient transfection and stable clone selection. HeLa cells (BCRC-60005), characterized by STR DNA profiling analysis, were purchased from BCRC in 2014, and were used for immunofluorescence staining within 6 months after resuscitation of the cells. R- cells, a fibroblast-like IGF-1R knockout cell line derived from mouse embryos, were a generous gift from Dr. Renato Baserga (Thomas Jefferson University, Philadelphia, PA, USA), and we have reconfirmed the absence of IGF-1R expression in these cells. The cells were cultured in Dulbecco's modified Eagle's medium supplemented with 10% fetal calf serum. G-418 (Promega, Fitchburg, WI, USA) was used for the selection of stable clones.

### Plasmids and constructs

Grb10 cDNA was obtained by polymerase chain reaction and cloned into pcDNA3.1 vector after the HA epitope. Nedd4 cDNA (*Xenopus*) was cloned into pCMV-Tag2B after the Flag epitope (Stratagene, Agilent, Santa Clara, CA, USA). The wild-type GPC3 expression vector pcDNA-GPC3 and P26->30A (previously designated P25->29A) have been previously described [20]. The deleted GPC3 expression vector (∆GPC3) was constructed by removing an *Apa*I flanking fragment (residues 70 to 453) from pcDNA-GPC3 [20]. The pHA-ubiquitin (HA-Ub) vector was a gift from Dr. Chen Hungwen [62]. shGPC3 has been previously described [20].

### Antibodies and immune assays

The antibodies used in the present study included anti-GPC3 (1G12; Santa Cruz Biotechnology, Santa Cruz, CA, USA), anti-IGF1Rβ (#3027, Cell Signaling Technology, Danvers, MA, USA), anti-phospho-ERK1/2 (Cell Signaling Technology), anti-Grb10, anti-Nedd4, anti-c-Myc (Santa Cruz Biotechnology), anti-ubiquitin (P4D1; Santa Cruz Biotechnology), anti-tubulin (Sigma-Aldrich, St. Louis, MO, USA), anti-Grb2 (BD Transduction Laboratories, San Jose, CA, USA), anti-Flag (Institute of Biological Chemistry, Academia Sinica, Taipei, Taiwan), and anti-HA (New England Biolabs, Ipswich, MA, USA). Paraffin-embedded tissue sections were deparaffinized, and immunochemical staining was performed after antigen retrieval. Endogenous peroxidase activity was blocked with EnVision™ FLEX Peroxidase-Blocking Reagent (Dako, Agilent). For western blot analyses, tissues and cells were extracted with HNTG buffer (20 mM HEPES buffer pH 7.5, 150 mM NaCl, 0.1% Triton X-100, and 10% glycerol). For detecting phosph-ERK1/2, the cells were collected in phosphoprotein lysis buffer [20]. Immunoprecipitation was performed by treating 0.5–1 mg of the cell lysate in HNTG buffer with antibodies, and the precipitated proteins were subjected to western blot analysis. All experiments were at least duplicated, but only representative pictures were shown. Densitometry quantitation after normalization by the quantity of tubulin or Grb2 was also expressed as a fold-change relative to the control experiment.

### Cell treatments

For IGF-1 (Invitrogen, Thermo Fisher Scientific, Waltham, MA, USA) stimulation, the cells were starved in serum-free medium for 24 hrs and subsequently treated with 50 ng/ml IGF-1 for various duration, as indicated. For IGF-1R ubiquitination analysis, the cells were treated with 20 μM of MG132 for 30 min and subsequently with IGF-1 for 30 min. Cell viability was determined by trypan blue staining as previously described [63]. Stable clones and control cells (1 × 10^4^ cells) were plated in 35-mm dishes with complete medium. The next day, the medium was replaced with either serum-free medium or medium supplemented with 0.5% fetal calf serum. The cells were trypsinized, stained with trypan blue, and counted at 48-hr intervals for 6 days.

### Pulse-chase assays for IGF-1R stability and co-immunoprecipitation

The cells were cultured in serum-free, cysteine-free medium (Gibco) for 45 min and labeled in the same medium containing 0.08 mCi of [^35^S]Cys-Met (Promix; Amersham Biosciences) for 1 h. The medium was subsequently replaced with serum-free medium (SFM) (time 0) containing IGF-I (20 ng/ml) and chased for 2, 4, and 6 h. Lysates from each time point were immunoprecipitated with IGF-1Rβ and loaded onto SDS-polyacrylamide gels. Densitometric analysis was performed using a BAS-1500 PhosphorImager.

### Confocal microscopy and other methods

After transfection, the cells were cultured in serum-free medium, stained with GPC3 antibody, and subsequently stimulated with 50 ng/ml of IGF-1 for 10 min. The cells were treated with 0.5% Triton X and fixed with 4% paraformaldehyde, and subsequently stained with anti-Grb10. The secondary antibodies were conjugated with Alexa 488 (green) for GPC3 and Alexa 594 (red) for Grb10, and then imaged by confocal laser scanning microscope. To assay oncogenicity in nude mice, 1 × 10^7^ cells in 200 μl of DMEM were injected into the flank of 4–5-week-old nude mice. Animal experiments were carried out using protocols approved by the Department of Medical Education and Research, Kaohsiung Veterans General Hospital, Taiwan (IACUC No. vghks-103-A018). To assay IGF-1R mRNA, the primers were mIGF1R-F, 5′-CGCTTGCCCTAAAACTGAAG-3′, and mIGF1R-R, 5′-GAGGGAGGTAGCCTGAATCC-3′. For actin, the primers were: actin F, 5′-TGCCTTAGGGTTGCAGG GGG-3′ and actin R, 5′-GTGGGCCCTCTAGGCA CCA-3′.

## SUPPLEMENTARY MATERIALS


