# Supplementary material for: Glypican-3 induces oncogenicity by preventing IGF-1R degradation, a process that can be blocked by Grb10
Source: Oncotarget. 2017 Jul 6;8(46):80429–42. doi: 10.18632/oncotarget.19035 (PMC5655209; doi:10.18632/oncotarget.19035)
Supplement: Supplementary file 1 [file oncotarget-08-80429-s001.pdf]

Glypican-3 induces oncogenicity by preventing IGF-1R degradation, a process that can be blocked by Grb10

Supplementary Materials

HepG2

Purchased from ATCC (HB-8065) in 2003
Reauthentication Oct 16, 2013 by STR DNA Profiling Analysis
Results: Similar to previous prescription

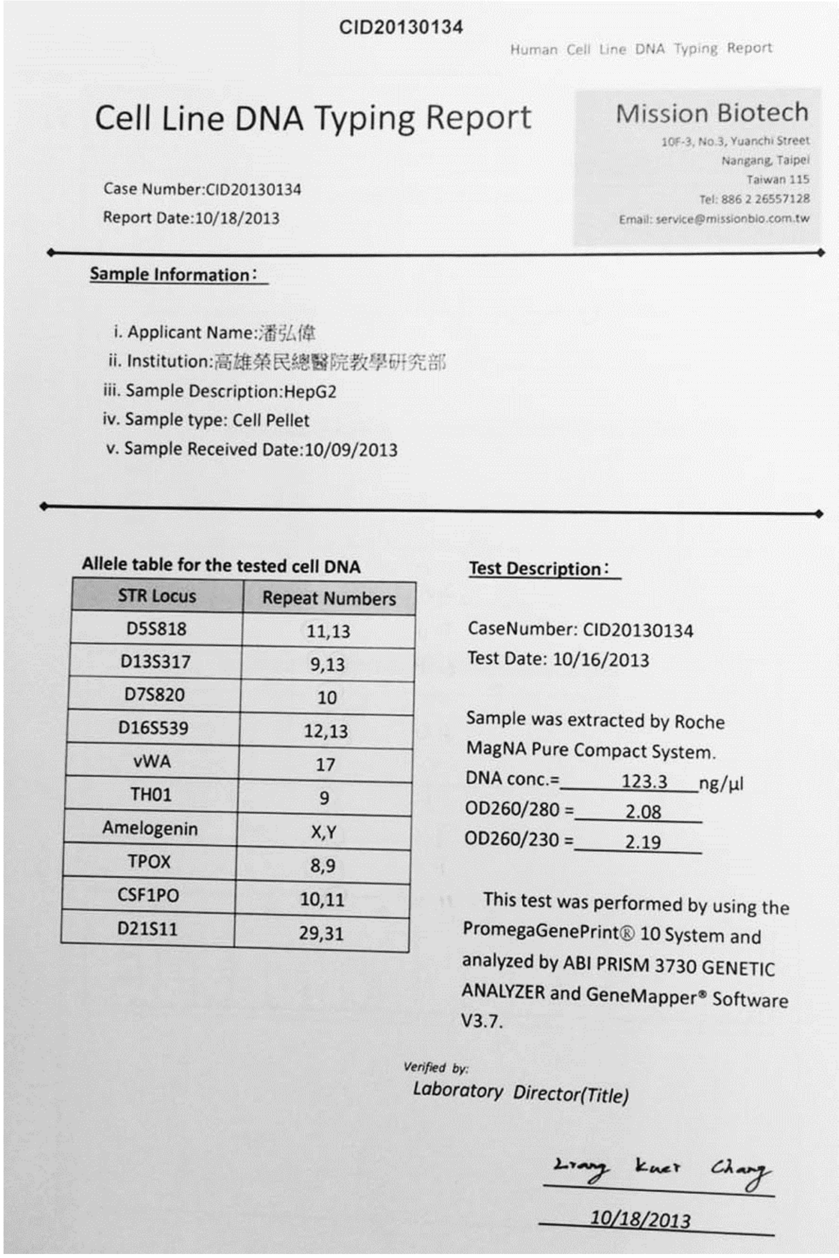

CID20130134

Human Cell Line DNA Typing Report

Cell Line DNA Typing Report

Mission Biotech
10F-3, No.3, Yuanchi Street
Nangang, Taipei
Taiwan 115
Tel: 886 2 26557128
Email: service@missionbio.com.tw

Case Number:CID20130134
Report Date:10/18/2013

Sample Information:

i. Applicant Name:潘弘偉
ii. Institution:高雄榮民總醫院教學研究部
iii. Sample Description:HepG2
iv. Sample type: Cell Pellet
v. Sample Received Date:10/09/2013

Allele table for the tested cell DNA

STR Locus	Repeat Numbers
D5S818	11,13
D13S317	9,13
D7S820	10
D16S539	12,13
vWA	17
TH01	9
Amelogenin	X,Y
TPOX	8,9
CSF1PO	10,11
D21S11	29,31

Test Description:

CaseNumber: CID20130134
Test Date: 10/16/2013

Sample was extracted by Roche MagNA Pure Compact System.
DNA conc.= 123.3 ng/μl
OD260/280 = 2.08
OD260/230 = 2.19

This test was performed by using the PromegaGenePrint® 10 System and analyzed by ABI PRISM 3730 GENETIC ANALYZER and GeneMapper® Software V3.7.

Verified by:
Laboratory Director(Title)

10/18/2013

HuH-7

Purchased from JCRB (JCRB0403) in 2005
Reauthentication Oct 15, 2013 by STR DNA Profiling Analysis
Results: Similar to previous prescription

Cell Line DNA Typing Report

Mission Biotech
10F-3, No.3, Yuanchi Street
Nangang, Taipei
Taiwan 115
Tel: 886 2 26557128
Email: service@missionbio.com.tw

Case Number:CID20130125

Report Date:10/15/2013

Sample Information:

i. Applicant Name:鄭威 Cheng Wei
ii. Institution:衛生福利部基隆醫院
Kee-Lung Hospital, Ministry of Health and Welfare
iii. Sample Description:Huh7
iv. Sample type: Cell Pellet
v. Sample Received Date:10/03/2013

Allele table for the tested cell DNA

STR Locus	Repeat Numbers
D5S818	11,12
D13S317	9,10,11,13
D7S820	10,11
D16S539	10,12,13
vWA	16,17,18
TH01	7,9
Amelogenin	X,Y
TPOX	8,9,11
CSF1PO	10,11
D21S11	29,30,31

Test Description:

CaseNumber: CID20130125
Test Date: 10/09/2013

Sample was extracted by Roche MagNA Pure Compact System.
DNA conc.= 208.6 ng/μl
OD260/280 = 2.01
OD260/230 = 2.30

This test was performed by using the PromegaGenePrint® 10 System and analyzed by ABI PRISM 3730 GENETIC ANALYZER and GeneMapper® Software V3.7.

Verified by:
Laboratory Director(Title)

Liang Kuei Chang

10/15/2013

PLC-PRF-5

Purchased from ATCC (CRL-8024) in 2003
Reauthentication Oct 15, 2013 by STR DNA Profiling Analysis
Results: Similar to previous prescription

Cell Line DNA Typing Report

Mission Biotech
10F-3, No.3, Yuanchi Street
Nangang, Taipei
Taiwan 115
Tel: 886 2 26557128
Email: service@missionbio.com.tw

Case Number:CID20130124
Report Date:10/15/2013

Sample Information：

i. Applicant Name:鄭威 Cheng Wei
ii. Institution:衛生福利部基隆醫院
Kee-Lung Hospital, Ministry of Health and Welfare
iii. Sample Description:PLC
iv. Sample type: Cell Pellet
v. Sample Received Date:10/03/2013

Allele table for the tested cell DNA

STR Locus	Repeat Numbers
D5S818	12,13
D13S317	8,11,12
D7S820	9,10,12
D16S539	12,13
vWA	16,18,19
TH01	7,8,9.3
Amelogenin	X,Y
TPOX	8,11
CSF1PO	10,11,12
D21S11	29,30,32.2

Test Description：

CaseNumber: CID20130124
Test Date: 10/09/2013

Sample was extracted by Roche MagNA Pure Compact System.
DNA conc.= 126.8 ng/µl
OD260/280 = 2.00
OD260/230 = 2.25

This test was performed by using the PromegaGenePrint® 10 System and analyzed by ABI PRISM 3730 GENETIC ANALYZER and GeneMapper® Software V3.7.

Verified by:
Laboratory Director(Title)

Liang Kuei Chang

10/15/2013

HEK293T

Purchased from ATCC (CRL-3216) in 2003
Reauthentication Jan 14, 2014 by STR DNA Profiling Analysis
Results: Similar to previous prescription

MB MISSION BIOTECH
明欣生物科技有限公司
台北市南港區園區街 3 號 10 樓之 1
Tel: 02-26557128
Email: genomics@missionbio.com.tw
http://www.missionbio.com.tw

Cell Line DNA Typing Report

Customer Details			
**Contact Name**	鄭威	**Principal Investigator**	鄭威
**Department**	衛生福利部基隆醫院解剖病理科	**Phone**	(02)247292525-5213

Sample Details			
**Sample Name**	293T	**Sample Received**	01/13/2014
**Sample Type**	Cell	**Sample Analyzed**	01/24/2014

Report	
**Genomic DNA Extraction**	☐ No<br>■ Yes. Genomic DNA was extracted by Roche MagNA Pure Compact System and quantified by Nanodrop.
**Analysis Reagent**	**GenePrint® 10 System**
**Analysis Platform**	**ABI PRISM 3730 Genetic Analyzer and GeneMapper® Software V3.7**
**Analysis Result**	(see table below)

STR Locus	Repeat Numbers
D21S11	28 , 29 , 30.2
TH01	7 , 9.3
TPOX	12 , 12
vWA	17 , 19 , 20
Amelogenin	X , X
CSF1PO	11 , 12
D16S539	9 , 10 , 14
D7S820	10 , 11
D13S317	11 , 12 , 14
D5S818	8 , 9
